# Extracellular localization of galectin-3 has a deleterious role in joint tissues

**DOI:** 10.1186/ar2130

**Published:** 2007-02-27

**Authors:** Audrée Janelle-Montcalm, Christelle Boileau, Françoise Poirier, Jean-Pierre Pelletier, Mélanie Guévremont, Nicolas Duval, Johanne Martel-Pelletier, Pascal Reboul

**Affiliations:** 1 Unité de Recherche en Arthrose, Centre de Recherche de l'Université de Montréal (CRCHUM), Montréal, Québec, H2L 4M1, Canada; 2 Universités Paris 6 et Paris 7, Institut Jacques Monod, CNRS UMR 7592, Place Jussieu, 75251 Paris Cedex 05, France; 3 Pavillon des Charmilles, boulevard des Laurentides, Vimont, Québec H7M 2Y3, Canada

## Abstract

In this study we examine the extracellular role of galectin-3 (gal-3) in joint tissues. Following intra-articular injection of gal-3 or vehicle in knee joints of mice, histological evaluation of articular cartilage and subchondral bone was performed. Further studies were then performed using human osteoarthritic (OA) chondrocytes and subchondral bone osteoblasts, in which the effect of gal-3 (0 to 10 μg/ml) was analyzed. Osteoblasts were incubated in the presence of vitamin D_3 _(50 nM), which is an inducer of osteocalcin, encoded by an osteoblast terminal differentiation gene. Genes of interest mainly expressed in either chondrocytes or osteoblasts were analyzed with real-time RT-PCR and enzyme immunoassays. Signalling pathways regulating osteocalcin were analyzed in the presence of gal-3. Intra-articular injection of gal-3 induced knee swelling and lesions in both cartilage and subchondral bone. On human OA chondrocytes, gal-3 at 1 μg/ml stimulated ADAMTS-5 expression in chondrocytes and, at higher concentrations (5 and 10 μg/ml), matrix metalloproteinase-3 expression. Experiments performed with osteoblasts showed a weak but bipolar effect on alkaline phosphatase expression: stimulation at 1 μg/ml or inhibition at 10 μg/ml. In the absence of vitamin D_3_, type I collagen alpha 1 chain expression was inhibited by 10 μg/ml of gal-3. The vitamin D_3_induced osteocalcin was strongly inhibited in a dose-dependent manner in the presence of gal-3, at both the mRNA and protein levels. This inhibition was mainly mediated by phosphatidylinositol-3-kinase. These findings indicate that high levels of extracellular gal-3, which could be encountered locally during the inflammatory process, have deleterious effects in both cartilage and subchondral bone tissues.

## Introduction

Osteoarthritis (OA) accounts for 40% to 60% of degenerative illnesses of the musculoskeletal system. On the whole, approximately 15% of the population suffers from OA. Of these, approximately 65% are 60 years of age and over. The high incidence of this illness is rather disturbing since its frequency increases gradually with the aging of the population.

It is well known that age is a primary risk factor for the development of OA, but the mechanisms by which aging contributes to an increased susceptibility to OA are poorly understood [1]. The end point of OA is cartilage destruction, which impairs joint movement and causes pain. In knee joints, the cartilage destruction is associated with and/or preceded by subchondral bone alterations [2]. Joint destruction is also associated with joint inflammation, where the synovial membrane plays a key role [3]. The chronological events of these phenomena are still debated in the literature. However, because of the complexity of the disease, its initiation could occur via any of these tissues, although inflammation of the synovial membrane is less likely to be a primary cause. In OA, it would appear that both cartilage and subchondral bone are altered extracellularly [4-7]. The age-related changes in chondrocytes result in a metabolic and phenotypic decline, triggering chondrocytes to be less responsive to growth factor stimulation and more prone to catabolic stimulation. This phenomenon could be the result of biomechanical forces as well as biological sources, such as cycles of hypoxia, the presence of reactive oxygen species, accumulation of advanced glycation end products and the effects of inflammatory cytokines [8-11]. Indeed, clinically detectable joint inflammation may predict a worse radiological outcome in OA [12].

Mechanisms by which synovitis exacerbates structural damage in OA are complex. Synovitis induces alterations in chondrocyte function and in subchondral bone turnover and enhances angiogenesis [13,14]. Cytokines, such as interleukin-1β and tumour necrosis factor-α, and growth factors are mainly responsible for these processes. However, another factor, galectin-3 (gal-3), can be markedly present in OA synovial tissue during inflammatory phases, in which leukocyte infiltration occurs [15]. These findings underline the potential deleterious role of gal-3 at the pannus level, where activated macrophages, a type of cell belonging to the leukocyte population able to secrete up to 30% of their gal-3, are present [3,16,17]. This indicates that gal-3 could be found extracellularly in the joint.

The exact role of gal-3 in articular tissues is not yet known. It is a soluble animal lectin of 30 kDa that preferentially recognizes lactosamine and N-acetyllactosamine structures [18,19]. Intracellularly, gal-3 is involved in a variety of processes, including RNA splicing [20], differentiation [21], and apoptosis [22]. Extracellularly, it is involved in cell-cell [23,24] or cell-matrix interactions [25-28]. Our recent work reported the capacity of normal and OA human chondrocytes to synthesize gal-3, with an increased expression level in human OA articular cartilage [29].

In the present study, we further investigate the role of extracellular gal-3 in joint tissues. To this end, we first examined its *in vivo *effect in mice having received an intra-articular injection of gal-3, and further investigated its effect on cells from two OA articular tissues: cartilage and subchondral bone.

## Materials and methods

### Intra-articular injection of galectin-3 in mice

Six-week-old 129c/c mice were housed in wire cages in animal rooms with controlled temperature, humidity, and light cycles. Mice were allowed food and water *ad libitum*. Recombinant human gal-3 (rh-gal-3) was prepared in our laboratory and sterilized on a 0.2 μm filter. As the amino acid sequence of rh-gal-3 shows 85% identical homology and 91% positive homology with murine gal-3, we injected rh-gal-3 into the knees of wild-type mice. Mice were distributed into 4 groups receiving 100 ng, 1 μg or 10 μg of gal-3 or vehicle (PBS) alone according to previous established protocols [30,31]. After being anaesthetized with isoflurane, a skin incision was performed on each knee and a single injection of gal-3 or PBS administered under the patellar ligament using a Hamilton syringe with a 26G^3/8 ^intradermal needle. The day of injection was considered day 0 (D0); the animals were sacrificed 4 days after the injection. The study was performed according to the Canadian Council on Animal Care regulations and was approved by the Animal Care Committee of the University of Montreal Hospital Centre.

### Knee joint swelling calculation

Animals were examined daily and knee diameter was measured using a digital calliper (model #2071M, Mitutoyo Corporation, Kawasaki, Japan) as described by Williams and colleagues [32]. The swelling corresponded to the difference between joint diameter measured every day and joint diameter prior to the injection.

### Cartilage histological grading

Histological evaluation was performed on the sagittal sections of the mouse knees removed at D4. Specimens were dissected, fixed in TissuFix #2 (Laboratoires Gilles Chaput, Montreal, QC, Canada), decalcified in RDO Rapid Decalcifier for bone (Apex Engineering, Plainfield, IL, USA), and embedded in paraffin. Serial sections (5 μm) were stained with safranin O and toluidine blue. The modifications in cartilage and subchondral bone were graded on a scale of 0 to 20 by two blinded, independent observers using a histological scale modified from Mankin and colleagues [33]. This scale was used to evaluate the severity of modifications based on the loss of staining with toluidine blue (scale 0 to 4), cellular changes (scale 0 to 4), surface/structural changes in cartilage (scale 0 to 5), structure of the deep zone of cartilage (scale 0 to 4), and subchondral bone remodelling (scale 0 to 3). Scoring was based on the most severe histological changes within each cartilage and subchondral bone section.

### Subchondral bone morphometry

The sections (5 μm) of each specimen were subjected to safranin O staining, as previously described [34]. A Leica DMLS microscope (Leica, Weitzlar, Germany) connected to a personal computer (Pentium III, using Image J software, V.1.27, NIH, USA) was used to perform the subchondral bone morphometry analysis. The subchondral bone surface (μm) was measured on each slide in two 500 μm × 250 μm boxes, using as the upper limit, the calcified cartilage/subchondral bone junction as previously described [34]. Two measurements were done and averaged for each section.

### Human osteoarthritis specimens

Femoral condyles and tibial plateaus were obtained from 15 OA patients (9 female and 6 male; aged 67 ± 9 years) following total knee arthroplasty. All patients were evaluated by a certified rheumatologist and, based on the criteria developed by the American College of Rheumatology Diagnostic Subcommittee for OA [35], were diagnosed as having OA. This procedure was approved by the Ethics Committee of the University of Montreal Hospital Centre.

### Human chondrocyte culture

Chondrocytes were released from the articular cartilage by sequential enzymatic digestion at 37°C, as previously described [36,37] and cultured in DMEM (Invitrogen, Burlington, ON, Canada) supplemented with 10% FBS (Invitrogen) and an antibiotic mixture (100 units/ml penicillin base, 100 μg/ml streptomycin base; Invitrogen) at 37°C in a humidified atmosphere of 5% CO_2_/95% air. Only first-passage cultured OA chondrocytes were used in the study. OA chondrocytes were seeded at 1 × 10^5 ^cells in 12 well plates in DMEM containing 10% FBS for 48 h; the medium was then replaced for 24 h by DMEM containing 0.5% FBS, after which the cells were incubated for 24 h in fresh media containing 0.5% FBS in the absence or presence of rh-gal-3 (0 to 10 μg/ml).

### Subchondral bone osteoblast culture

The overlying cartilage was removed from the tibial plateaus, and the trabecular bone tissue was dissected from the subchondral bone plate. Primary subchondral osteoblasts were released as previously described [38]. Briefly, subchondral bone samples were cut into small pieces of 2 mm^2 ^before sequential digestion in the presence of 1 mg/ml collagenase type I (Sigma-Aldrich, Oakville, ON, Canada) in DMEM without serum at 37°C for 30, 30, and 240 minutes. After being washed with the same medium, the digested subchondral bone pieces were cultured in DMEM containing 10% FBS. This medium was replaced every two days until cells were observed in the petri dishes. At confluence, cells were passaged once in 12- or 24-well plates in DMEM containing 10% FBS. Experiments were performed in DMEM containing 0.5% of charcoaled FBS with or without 50 nM 1,25 [OH]_2 _D_3 _(1,25-dihydroxycholecalciferol; vitamin D_3_) in combination or not with gal-3. To evaluate signalling pathways involved in vitamin D_3_-stimulated osteocalcin production that are inhibited by gal-3, cells were pre-incubated for 2 h with specific inhibitors and then incubated for 22 h in the presence of the inhibitors and vitamin D_3 _in combination or not with gal-3. The inhibitors used were KT5720 (inhibitor of protein kinase A; final concentration 2 μM), KT5823 (inhibitor of protein kinase G; final concentration 2 μM), Genistein (broad inhibitor of tyrosine kinase; final concentration 20 μM), Taxifolin (an antioxidant flavonoid; final concentration 1 μM), wortmannin (inhibitor of phosphatidylinositol 3-kinase (PI 3-kinase); final concentration 250 nM), PD98059 (inhibitor of mitogen-activated protein kinase kinase-1 (MEK-1) activation; final concentration 10 μM), and SB202190 (inhibitor of p38 mitogen-activated protein kinase; final concentration 2 μM). All inhibitors were purchased from Calbiochem (San Diego, CA, USA).

### Real time RT-PCR

RNA extraction and real time RT-PCR were performed as previously described [29]. Primers for the genes encoding a disintegrin and metalloproteinase with thrombospondin type 1 motif (ADAMTS)-5 (aggrecanase-2), matrix metalloproteinase (MMP)-3 (stromelysin), osteocalcin, alkaline phosphatase and type I collagen α1 chain were synthesized by Invitrogen (Table 1). Data analysis was carried out using the Gene Amp 5700 Sequence Detector System software (Applied Biosystem, Foster City, CA, USA) and values normalized to the ribosomal subunit 18S. Specific primers for type I collagen α1 chain were designed using Primer3 software [39].

### Osteocalcin determination

The assay measured only intact human osteocalcin and was performed on human osteoblast-conditioned media using a specific enzyme immunoassay (EIA) kit with a sensitivity of 0.5 ng/ml (Biomedical Technologies Inc., Stoughton, MA, USA).

### Protein determination

Cells were lysed in 0.5% sodium dodecylsulfate and proteins quantified with the bicinchoninic acid assay [40].

### Statistical analysis

Data are expressed as mean ± SEM or median (range). Statistical analyses were the Mann-Whitney U and the two-tailed Student's *t*-tests for animal experiments and cell culture, respectively. Results of *p *< 0.05 were considered significant.

## Results

### Intra-articular injection of galectin-3

**Figure 1:**
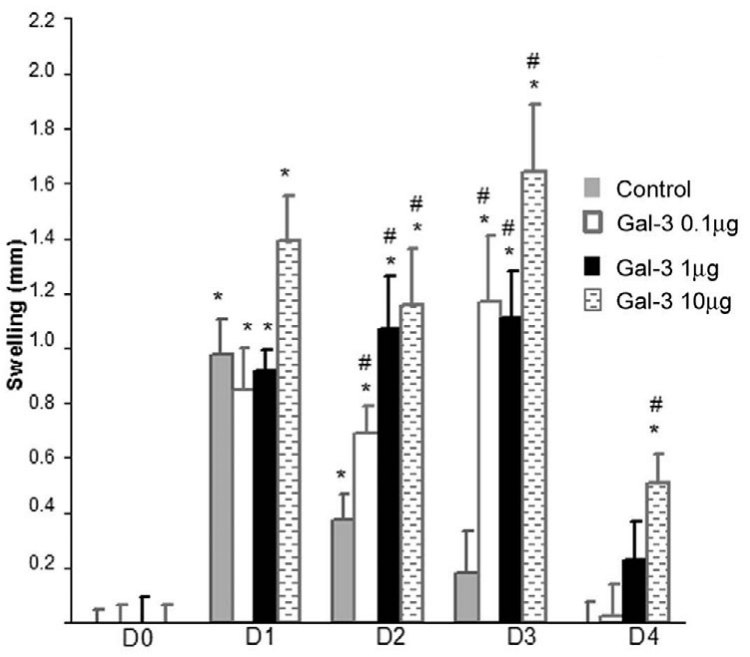
Mouse knee swelling measurement. Galectin-3 (gal-3) was injected in both knees. Animals were examined daily and knee diameter measured using a digital calliper as described in Materials and methods. The swelling corresponded to the difference between joint diameter measured every day and joint diameter prior to the injection (D0). D0 was given the value of 0. Control (Ctl): injection of PBS. Each group contained four animals. *p versus same conditions as D0; ^#^*p *versus control of the corresponding day.

As Ohshima and colleagues [15] showed that gal-3 was markedly present in OA synovial tissues during the inflammatory phase and could be recovered in the synovial fluid, we explored the potential extracellular role of gal-3. We injected gal-3 (0.1, 1, and 10 μg) into the knee joints of mice. To evaluate the potential role of gal-3 in the inflammation process we first determined if this molecule induces joint swelling. Data show that the vehicle alone (control) induced a joint swelling at D1 (*p *≤ 0.0002 versus D0) (Figure 1). Although joint swelling at D2 was significantly lower compared to D1 (*p *< 0.005), a significant difference was still seen when D2 was compared to D0 (*p *< 0.004). Values gradually returned to the basal conditions. Gal-3 exacerbated and extended the swelling; thus, at D2, gal-3 injections of 0.1, 1, and 10 μg significantly induced higher swelling than the vehicle alone (*p *< 0.05, *p *< 0.004 and *p *< 0.002, respectively). This effect was sustained the third day post-injection (*p *< 0.006 for 0.1 μg, *p *< 0.002 for 1 μg, *p *< 0.0001 for 10 μg). Finally, at D4, values tended to return to those of the control group, although gal-3-induced joint swelling was still statistically significant (*p *< 0.006) with the highest gal-3 concentration used.

**Figure 2:**
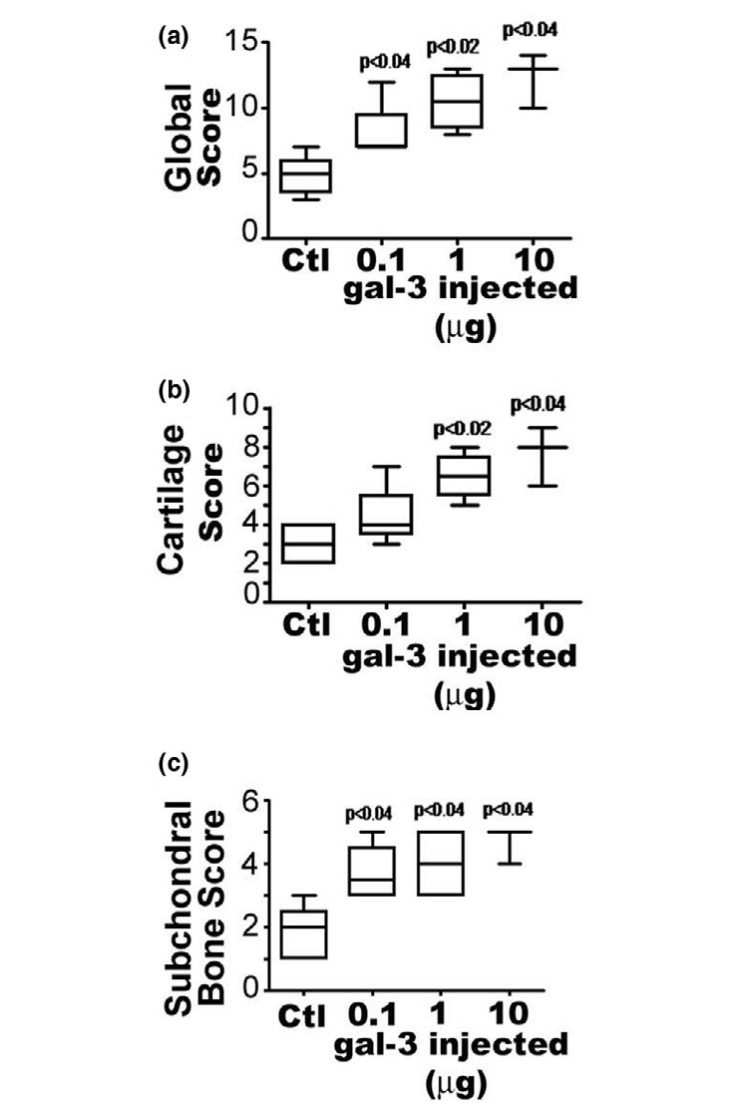
Histological score for mice four days after intra-articular galectin-3 (gal-3) injection. **(a) **Total score, **(b) **cartilage score and **(c) **bone histomorphometric score. Data are expressed as median and (range) and are presented in box plot, where the boxes represent the 1st and 3rd quartiles, the line within the box represents the median, and the lines outside the box represent the spread of the values. Control (Ctl): mice injected with PBS. *P *versus control group; *n *= four animals per group.

**Figure 3:**
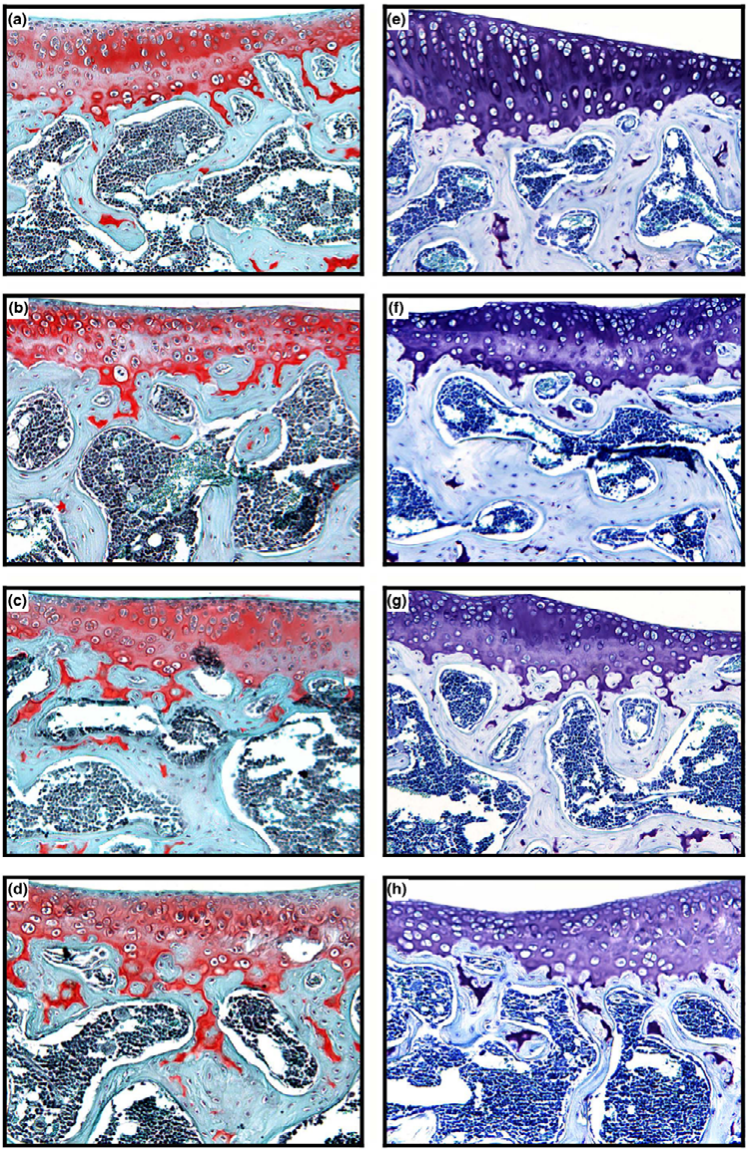
Representative histological sections of specimens from mice stained with **(a-d) **safranin O or **(e-h) **toluidine blue (magnification ×100). Control group (a,e). Mice were injected with 0.1 μg (b,f), 1 μg (c,g), or 10 μg (d,h) galectin-3.

Furthermore, we investigated the effect of gal-3 on cartilage and subchondral bone using histological means. The global histological score (median and (range)) in the control group was 5.0 (3.5 to 6.0) whereas it reached 9.5 (7.0 to 12.5) (*p *< 0.04 versus control), 10.5 (8.5 to 12.5) (*p *< 0.02 versus control) and 13 (*p *< 0.04 versus control) in the gal-3-injected group with 0.1, 1, and 10 μg gal-3, respectively (Figure 2a). The cartilage score in the control group was 3.0 (2.0 to 4.0) whereas it reached 4.0 (3.5 to 5.5), 6.5 (7.5 to 5.5) (*p *< 0.02 versus control) and 8 (*p *< 0.04 versus control) in the gal-3-injected group with 0.1, 1, and 10 μg gal-3, respectively (Figure 2b). The subchondral score in the control group was 2.0 (1.0 to 2.5) whereas it reached 3.5 (3.0 to 4.5) (*p *< 0.04 versus control), 4.0 (3.0 to 5.0) (*p *< 0.04 versus control) and 5 (*p *< 0.04 versus control) in the gal-3-injected group with 0.1, 1, and 10 μg gal-3, respectively (Figure 2c). Therefore, both the cartilage parameters (structure/surface, cellularity, and toluidine blue staining) and the subchondral bone surface were modified by the gal-3 injection (Figure 3). These modifications are illustrated in Figure 3, which shows changes in the surface, in cellularity and remodelling of the deep layers in the presence of gal-3 (left panel (b-d)) compared to the control group. Destaining and modification of cell columns were also noticed in the presence of gal-3 (left panel (f-h)) compared to the control group.

### Effects of galectin-3 on chondrocytes and osteoblasts

#### Effect of galectin-3 on ADAMTS-5 and MMP-3 in human OA chondrocytes

**Figure 4:**
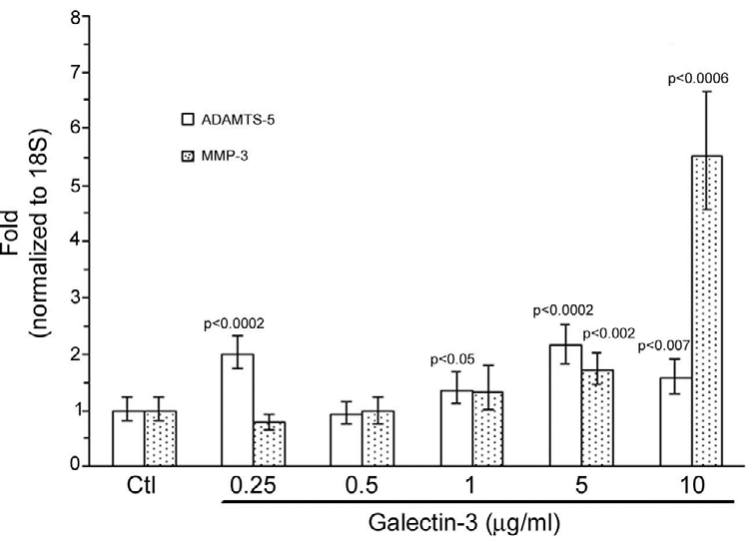
Effects of exogenous galectin-3 (gal-3) on human osteoarthritis chondrocytes. Chondrocytes were treated with increasing concentrations of recombinant human gal-3. Both ADAMTS-5 and matrix metalloproteinase (MMP)-3 expression were analyzed by real time RT-PCR. *P *versus control (Ctl); *n *= 5.

*In vivo *data strongly suggest that extracellular gal-3 affects both chondrocytes and osteoblasts. We therefore further explored the effects of gal-3 on human OA cells and examined enzymes and markers of these cells. For chondrocytes, two major enzyme systems were evaluated: ADAMTS-5 and MMP-3. Data show that human OA chondrocytes incubated with rh-gal-3 for 24 h increased ADAMTS-5 expression in a biphasic mode. Indeed, it is interesting to note that this gene is very sensitive to gal-3 since a concentration as low as 0.25 μg/ml is sufficient to significantly enhance its expression. Another peak of stimulation was obtained with a concentration of 5 μg/ml (Figure 4). MMP-3 expression was only slightly induced at low concentration and significance was reached at 5 μg/ml with a major increase obtained at 10 μg/ml (Figure 4).

#### Effects of galectin-3 on osteoblastic markers in human OA subchondral bone osteoblasts

**Figure 5:**
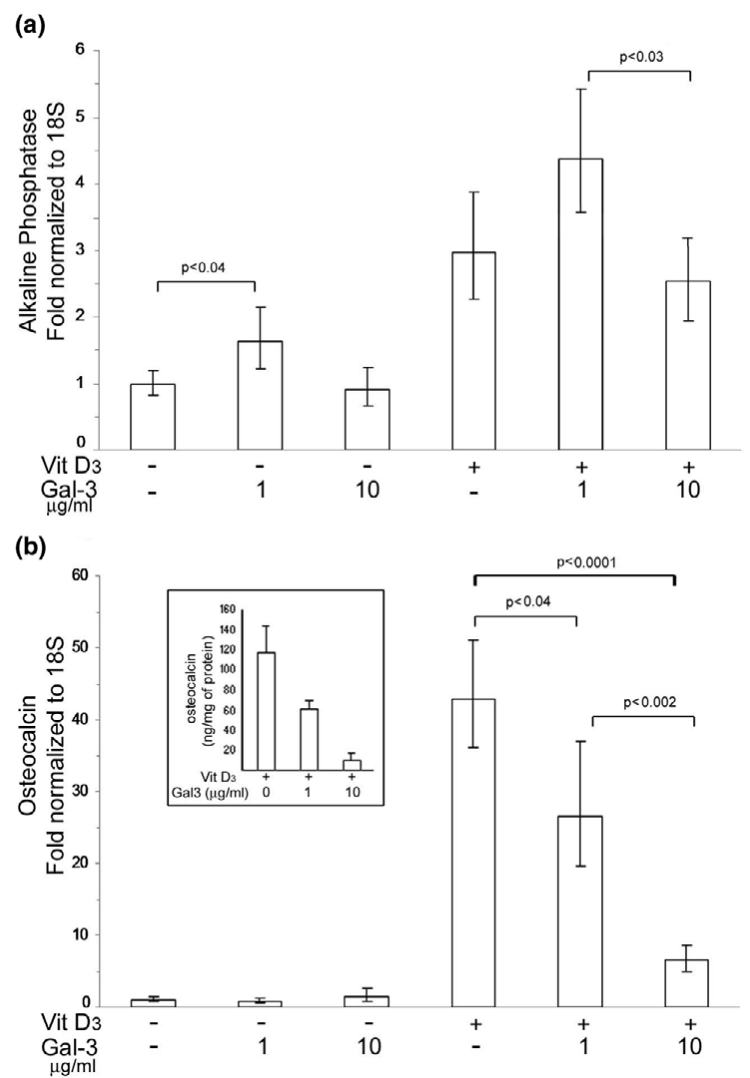
Effects of exogenous galectin-3 (gal-3) on markers of subchondral bone osteoblasts. Osteoblasts were treated with 1 or 10 μg/ml of recombinant human gal-3 in the presence or absence of vitamin D_3_. Both **(a) **alkaline phosphatase and **(b) **osteocalcin expression were analyzed by real time RT-PCR. Insert illustrates the protein level of osteocalcin. *N *= 4.

The effects of gal-3 on human osteoblasts were evaluated in the presence or absence of vitamin D_3_, which allows the terminal differentiation of these cells. Alkaline phosphatase expression was increased with gal-3 at 1 μg/ml (*p *< 0.04), but not at 10 μg/ml (Figure 5a). In contrast, the latter concentration triggered significantly lower alkaline phosphatase expression than 1 μg/ml (*p *< 0.04). Alkaline phosphatase, which is upregulated by vitamin D_3_, tended to be increased with gal-3 at 1 μg/ml (*p *< 0.07). A significant difference in alkaline phosphatase expression was found between osteoblasts treated with vitamin D_3 _in the presence of 1 μg/ml gal-3 and vitamin D_3 _in the presence of 10 μg/ml gal-3 (*p *< 0.03).

As previously described, in the absence of vitamin D_3_, osteocalcin expression was maintained at a minimal level, and gal-3 had no effect on osteocalcin expression (Figure 5b). In contrast, in the presence of vitamin D_3_, gal-3 induced a dose-dependent inhibition of osteocalcin expression. Indeed, vitamin D_3 _alone stimulated a 43-fold increase in osteocalcin expression compared to the basal level, whereas the addition of either 1 μg/ml gal-3 or 10 μg/ml gal-3 with vitamin D_3 _induced osteocalcin expression to only 26.5 (*p *< 0.04) and 6.5 (*p *< 0.0001) times the basal level, respectively. These results were confirmed at the protein level by analyzing osteocalcin concentration in conditioned media using an EIA. Osteocalcin production was inhibited by around 40% and 85% at gal-3 concentrations of 1 and 10 μg/ml, respectively (Figure 5b, insert). We verified the inhibition of osteocalcin production with a commercially available rh-gal-3 (R&D Systems, Minneapolis, MN, USA). Results obtained from these experiments were 138.7 ± 21.2 (mean ± SEM; ng/mg protein; *n *= 3) for osteoblasts treated with vitamin D_3 _alone, 67.6 ± 7.9 for those treated with 1 μg/ml rh-gal-3 in the presence of vitamin D_3 _and 2.4 ± 0.9 for cells treated with 10 μg/ml rh-gal-3 in the presence of vitamin D_3_. In addition, we made a truncated isoform of gal-3 (Gly108 to Ile249) corresponding to the carbohydrate recognition domain (CRD). This truncated isoform is known to be incapable of multimerizing and it is unable to reproduce the effects of whole gal-3. Results obtained with an EIA were 130.2 ± 16.5 (mean ± SEM; ng/mg protein; *n *= 7) for osteoblasts treated with vitamin D_3 _alone, 158.5 ± 22.6 for those treated with 1 μg/ml CRD in the presence of vitamin D_3 _and 163.4 ± 26.1 for those treated with 5 μg/ml CRD in the presence of vitamin D_3_. As expected, CRD was not able to downregulate the osteocalcin production.

**Figure 6:**
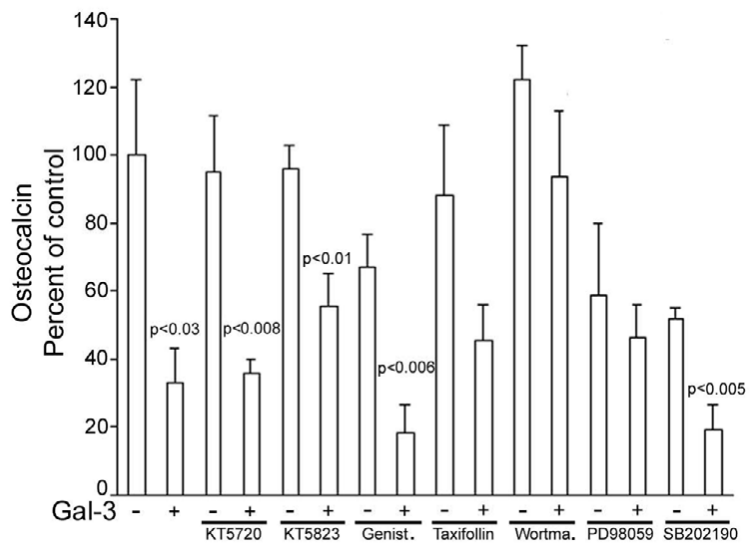
Signalling pathways of inhibition by galectin-3 (gal-3) of vitamin D_3_-stimulated osteocalcin production. Osteoblasts were treated with 5 μg/ml of recombinant human gal-3 in the presence of vitamin D_3 _and osteocalcin was determined. Inhibitor concentrations were: KT5720, 2 μM; KT5823, 2 μM; Genistein (Genist.), 1 μM; Taxifollin, 1 μM; wortmannin (Wortma.), 250 nM; PD98059, 10 μM; and SB202190, 2 μM. *P versus the autologous control; *n *= 5.

As 10 μg/ml gal-3 almost entirely inhibited osteocalcin production, we further examined the signalling cascades of gal-3 inhibition of vitamin D_3_-stimulated osteocalcin production with 5 μg/ml gal-3, which resulted in an inhibitory effect closer to 50% (Figure 6). Vitamin D_3_-stimulated osteocalcin production tended to be inhibited by genistein (35%) and SB202190 (40%), indicating that tyrosine kinases and p38 mitogen-activated protein kinase may be slightly involved (Figure 6). However, the addition of gal-3 in the presence of these inhibitors still induced further inhibition, which was statistically significant (*p *< 0.006 and *p *< 0.005, respectively), indicating that gal-3 did not induce these pathways. The combination of gal-3 with either KT5720 or KT5823 also significantly inhibited osteocalcin production compared to their respective controls (*p *< 0.008 and *p *< 0.01, respectively), indicating that neither protein kinase A nor protein kinase G are involved in gal-3-inhibited osteocalcin production. This result was confirmed by the fact that gal-3 alone and gal-3 in the presence of KT5823 did not produce results with a significant difference. In contrast, PD98059 prevented further inhibition of osteocalcin production by gal-3. This result indicates that Erk1/Erk2 kinases are also involved to some extent in gal-3 signalling transduction. Taxifollin, an antioxidant flavonoid, also seemed to prevent gal-3 inhibition of osteocalcin production, but this inhibitor had the weakest effect. The most spectacular result was obtained with an inhibitor of PI 3-kinase, wortmannin, which totally prevented the inhibition of osteocalcin by gal-3.

**Figure 7:**
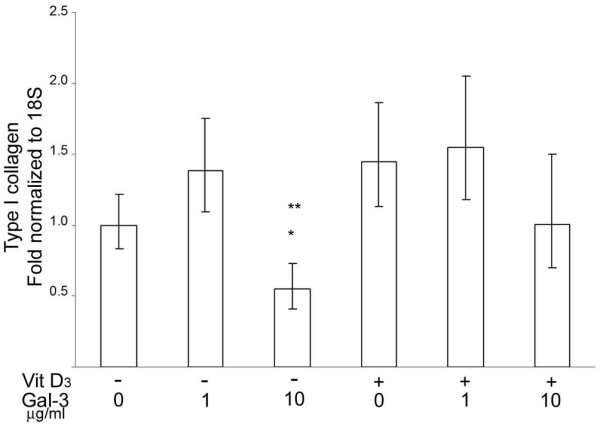
Effects of exogenous galectin-3 (gal-3) on type I collagen expression in osteoblasts. Osteoblasts were treated with 1 or 10 μg/ml of recombinant human gal-3 in the presence or not of vitamin D_3_. Collagen type I α1 chain expression was analyzed by real time RT-PCR. *P versus control (Ctl; without vitamin D3 or gal-3); **p versus 1 μg gal-3 alone; *n *= 4.

As type I collagen is the most abundant protein of the osteoid, we finally investigated whether gal-3 affects expression of the type I collagen α1 chain in subchondral bone osteoblasts. In the absence of vitamin D_3_, 10 μg/ml of gal-3 inhibited 50% of type I collagen α1 chain expression (*p *< 0.02) but this inhibitory effect was partly reversed by vitamin D_3 _(Figure 7).

## Discussion

In the present study, we show that extracellular gal-3 induced swelling and OA-like lesions in the knee joints of mice. These findings were confirmed by the experiments in which we demonstrated in human OA chondrocytes that gal-3 stimulated the expression of ADAMTS-5 and MMP-3, the main enzymes involved in proteoglycan degradation in cartilage. Furthermore, using human osteoblasts, we showed that gal-3 inhibited osteocalcin production, which is encoded by the most specific and latest gene expressed by differentiated osteoblasts.

Results obtained by Ohshima and colleagues [15] demonstrated that intra-articular production of gal-3 could occur in joints even during OA, and particularly during inflammatory phases. Very often, these phases lead to hyperplasia of the synovium, which may invade the joint space and adhere to cartilage, generating a pannus. This pannus is composed of very active cells such as leukocytes and, most importantly, macrophages, which are able to secrete high levels of gal-3 when they are activated. Therefore, we injected gal-3 into the knee joints of mice and evaluated the structural changes. We found that gal-3 induced a swelling that was sustained compared to injection of PBS alone. Moreover, gal-3 injection generated lesions that affected both cartilage and subchondral bone tissue.

It is interesting to note that two major enzymes responsible for proteoglycan degradation were stimulated by gal-3. This finding corroborates the *in vivo *data, in which cartilage presented with both alterations and fainter staining with toluidine blue in gal-3 injected mice. However, not all MMPs were stimulated by gal-3 in chondrocytes, since collagenase-3 (MMP-13) was unaffected (data not shown). In addition, the level of tissue inhibitor of MMP-1 (TIMP-1), a natural protein inhibitor produced by chondrocytes, also remained stable (data not shown). We show that ADAMTS-5 was more sensitive than MMP-3 to gal-3, since its expression was stimulated with very low concentrations of gal-3, unlike MMP-3, which required higher concentrations for stimulation. The regulation of ADAMTS-5 is crucial since it was recently demonstrated by two independent groups (using knock-out mouse models) that ADAMTS-5 is the major aggrecanase responsible for proteoglycan degradation in cartilage destruction [41,42]. On the other hand, we so far have no explanation for the rebound phenomenon observed for ADAMTS-5 stimulation with 1 μg/ml gal-3.

Gal-3 not only modulated chondrocyte-expressed genes but also those of osteoblasts. More particularly, production of osteocalcin, which is an osteoblastic marker [43], was strongly inhibited by gal-3. Furthermore, the multimerization of gal-3 is needed to induce this effect since the CRD, which is a truncated isoform of gal-3 lacking this property, has no effect. The membranous target recognized by gal-3 is still unknown in osteoblasts. However, among other targets, gal-3 is able to bind integrin β1. Interestingly, a recent study reported that the downregulation of integrin β1 with either small interfering RNA or blocking antibodies decreased the vitamin D_3_-stimulated osteocalcin level [44]. One hypothesis is that gal-3 may act, at least partially, by blocking integrin β1 at the osteoblast surface. Among different cascades of regulation involved in the inhibition of vitamin D_3_-stimulated osteocalcin levels, the PI 3-kinase appears to be a key enzyme. This could be related to the implication of integrins, since it has recently been shown that several biological functions of osteoblasts are regulated via the integrin/PI 3-kinase pathway [45,46].

Unlike osteocalcin, type I collagen α1 chain expression was downregulated only with a high gal-3 concentration. However, vitamin D_3 _prevented the inhibition of type I collagen expression. This latter finding raised the potential role of gal-3 in preventing osteoid matrix formation during the inflammatory process, particularly in individuals with low or depleted levels of vitamin D_3 _since it has been shown that vitamin D_3 _analogues have immunomodulatory effects [47].

## Conclusion

The presence of extracellular gal-3 in the vicinity of chondrocytes and osteoblasts causes deleterious effects by both downregulating the anabolic processes and upregulating the catabolic processes. In fact, this factor may participate in cartilage destruction and subchondral bone erosion, particularly during the highly inflammatory phases of OA.

## Abbreviations

ADAMTS-5 = a disintegrin and metalloproteinase with thrombospondin type 1 motif; CRD = carbohydrate recognition domain; D = day; DMEM = Dulbecco's modified Eagle's medium; EIA = enzyme immunoassay; FBS = fetal bovine serum; Gal-3 = galectin-3; MMP = matrix metalloproteinase; OA = osteoarthritis; PBS = phosphate buffered saline; PI 3-kinase = phosphatidylinositol 3-kinase; rh-gal-3 = recombinant human gal-3.

## Competing interests

The authors declare that they have no competing interests.

## Authors' contributions

AJM contributed to the *in vitro *study, analyzed the data and drafted the manuscript. CB participated to the animal study design, analyzed the data and drafted the manuscript. MG participated in the *in vitro *study. FP, JPP, ND, JMP, PR contributed to the study design. FP, JPP, JMP, PR contributed to the revision of the final manuscript.

## Figures and Tables

**Table 1:** Primers used for RT-PCR

Amplified gene product	Primers	Base pairs	Reference
ADAMTS-5	S: GGCATCATTCATGTGACAC AS: GCATCGTAGGTCTGTCCTG	364	
MMP-3	S: GAAAGTCTGGGAAGAGGTGACTCCAC AS: CAGTGTTGGCTGAGTGAAAGAGACCC	284	
Osteocalcin	S: CATGAGAGCCCTCACA AS: AGAGCGACACCCTAGAC	310	[48]
Alkaline phosphatase	S: TGCAGTACGAGCTGAACAG AS: TGAAGACGTGGGAATGGTC	267	
Type I collagen α1 chain	S: CCGAAGGTTCCCCTGGACGA AS: CGCCCTGTTCGCCTGTCTCA	252	
18S	S: GAATCAGGGTTCGATTCCG AS: CCAAGATCCAACTACGAGC	279	[29]

S, sense; AS, antisense.
